# Association Between HTLV-I Infection with Chronic Lupoid Leishmaniasis

**Published:** 2013-03

**Authors:** Fakhrozaman Pezeshkpoor, Seyed Abdol Rahim Rezaei, Abbas Shirdel, Mohammad Khajedaluee, Mansoreh Alizadeh, Mohammad Javad Yazdanpanah

**Affiliations:** 1 Research Centre for Skin Diseases and Cutaneous Leishmaniasis, Department of Dermatology, Faculty of Medicine, Mashhad University of Medical Sciences, Mashhad, Iran; 2 HTLV-I Foundation, Immunology Department, Mashhad University of Medical Sciences, Mashhad, Iran; 3 Department of Internal Medicine, Mashhad University of Medical Sciences, Mashhad, Iran; 4 Department of Social Medicine, Mashhad University of Medical Sciences, Mashhad, Iran

**Keywords:** Cutaneous lupoid leishmaniasis, Mashhad type 1 human T cell lymphotropic virus

## Abstract

***Objective(s):*** One of the different types of skin leishmaniasis is the Chronic Lupoid Leishmaniasis (CLL), which is caused by abnormal immune response. On the other hand, HTLV-I has been known to exist in some infectious diseases. Human T cell lymphotropic virus type1 (HTLV-I) and cutanous leishmaniasis exists endemically in Mashhad. The objective of this study was to evaluate the prevalence of HTLV-I in CLL patients.

***Materials and Methods:*** This cross sectional study involved 51 CLL patients admitted to cutaneous leishmaniasis clinics of Ghaem and Imam Reza hospitals in Mashhad, Iran. The blood samples were examined for serology tests through ELISA method.

***Results: ***The results of the experiments for evaluating the existence of HTLV-I in 51 patients under study in this research were proved to be negative.

***Conclusion: ***According to this pilot study, the distribution of HTLV-I in CLL patients is not higher than normal population.

## Introduction

Leishmaniasis consists of chronic human infections, developed by types of leishmaniasis parasites that are in turn the intracellular protozoan parasites.(1) cutaneous leishmaniasis is endemically found in different regions of Iran, including Mashhad.(2) CLL is caused by the recurrent papules around the scars of an old leshmaniasis (3). The exact etiology for the existence of such a leshmaniasis is still unknown. CLL is a clinical form of leshmaniasis. Despite the immune response to parasites and a heightened hypersensitive response, the body’s immune system will not be able to remove the parasite completely and a chronic granulomatous response continues to exist for a long period of time. (4-6) In 2011, the prevalence of HTLV-I in Mashhad was reported to be 2.12%.(7)

Due to the simultaneous spread of these two diseases in Mashhad on the one hand and the evidences of body’s immune system’s role in those diseases on the other hand, and in order to have a better understanding of CLL pathogens and HTLV-I infections, we decided to examine the HTLV-I infections in CLL patients.

## Materials and Method

In this cross-sectional pilot study, conducted in 2010-2011, 51 CLL patients were included in the examinations at cutaneous leishmaniasis clinics of Ghaem and Imam Reza Hospitals in Mashhad, Iran. The project started soon after obtaining the permission from Ethical Committee. The evidence for the existence of CLL in these patients was based on the primary samples of leishmaniasis lesions after one year and a present clinical form of leishmaniasis. In clinical cases, the lesions were the scars of the newly recovered leishmaniasis, along with the recurrent activation of papules or nodules around or on it. The primary diagnosis was mostly done through smear tests. The skin biopsies confirmed the data collected from the early indefinite diagnosis for 8 patients. After giving sufficient information about the objectives of the study to the patients and obtaining formal consent from them, (in the case of the children, the consent was obtained from their parents), the patients’ special questionnaire were filled. The questionnaire included some information such as the exact description of leishmaniasis, other diseases of the patient and risk factors in HTLV-I infection transmission. The patients’ blood samples were taken to the laboratory and were examined for serology tests through ELISA (Diapro kite, made in Italy) method. It was expected that in the case of positive results of ELISA test, the patient’s blood test should be further examined by PCR in order to test the RNA viruses and proviral load. 

In order to analyze the data, and to consider demographic characteristics, SPSS software version 16 and T-test and Chi-square nonparametric statistical tests were used.

## Results

Fifty-one patients were under study. The mean age was 21.37±19.1 years (Min=3 years, Max=70 years). 29 patients (56.9%) were female and 22 of them (43.1) were male. Fifteen patients had more than one lesion. Location of lesions in the majority of cases was on the face. Mean age of the lesions was 2.08 years in females and 4.2 years in males. ELISA tests for serology examinations were negative for all cases.

## Discussion

Cutaneous leishmaniasis exists endemically in Mashhad. One of the different types of cutaneous leishmaniasis is the chronic lupoid leishmaniasis which may be generated after infection with leishmaniasis tropica.(4) Chronic course is one of the specifications of this type of leishmaniasis. In this regard, leishmaniasis treatment is also an underlying issue, in a way that most routine and common medications failed to remove the injury.(3)

Moreover, the investigations about the HTLV-I related infectious diseases showed the wide spread of the complexities with infectious sources such as strongyloides(8), scabies, leprosy, herpes labial, seborrheic dermatitis. (9,10)

HTLV-I might be responsible forcreating disharmonies in body’s immune system through insinuating changes. Thus, it can reinforce the possibility of complications originating from the suppressive immune system and also those originating from the autoimmune system in other diseases. (11)

In addition to the above-mentioned issues with the main basis for this study is the fact that both HTLV-I and CLL exist endemically in Mashhad. In a study carried out in 1999 in Columbia, the coexistence of HTLV-I with cutaneous leishmaniasis (in three groups of severe, chronic and clinical infections) was examined and was subsequently rejected. (12) In the present study, too, serologic examination of HTLV-I through ELISA was reported to be negative for all patients.

In conclusion, it seems that the distribution of HTLV-I in CLL patients is not much higher than in normal cases 

It is obvious that focusing on the body immune system factors is not enough for investigating the CLL pathogenesis and other factors including parasite-inducing factors, environmental factors such as sunlight, UV rays and also host-related factors such as genetic factors, age etc should be taken into account.. However, CLL can is known to have multiple reasons. (13)
